# Electroacupuncture for treating depression-related insomnia: a systematic review and meta-analysis

**DOI:** 10.3389/fpsyt.2025.1610107

**Published:** 2025-07-08

**Authors:** Yu Gao, Tie Li, Qi Lu, Jiaxin Wang, Ying Wang, Long Wang

**Affiliations:** ^1^ Department of Acupuncture and Tuina, Changchun University of Chinese Medicine, Changchun, Jilin, China; ^2^ Department of acupuncture and moxibustion, Affiliated Hospital of Changchun University of Chinese Medicine, Changchun, Jilin, China; ^3^ Northeast Asian Institute of Chinese Medicine, Changchun University of Chinese Medicine, Changchun, Jilin, China

**Keywords:** electroacupuncture, depression, insomnia, systematic review, meta-analysis, randomized controlled trial

## Abstract

**Background:**

Depression is a major mental disorder worldwide, affecting over 264 million people. Approximately 50% of individuals with depression also experience insomnia. The treatment of depression may be complicated by comorbid insomnia. Electroacupuncture (EA) has been widely used in clinical practice, with substantial empirical evidence accumulated for its role in treating both depression and related insomnia. However, a systematic evaluation of its efficacy is lacking. This study seeks to assess the efficacy and safety of EA for treating insomnia related to depression.

**Methods:**

Web of Science, Sinomed, Cochrane Library, CNKI, Wanfang, Embase, and PubMed were searched up to November 2024 to select randomized controlled trials (RCTs) investigating EA for treating depression-related insomnia. The literature was selected and the data were extracted separately by two reviewers. The methodological quality of eligible RCTs was evaluated via the risk of bias 2 tool. Meta-analysis was carried out via Stata SE V.15.0 software.

**Results:**

14 studies involving 1342 individuals with depression-related insomnia were included. The results unraveled that EA was more effective in reducing PSQI scores in comparison to the interventions used in the controls (WMD = -2.757, 95% CI: -3.759 to -1.755). Moreover, EA demonstrated superior efficacy in lowering HAMD scores in contrast to the interventions used in the controls (WMD = -3.587, 95% CI: -4.992 to -2.182). Regarding lowering PSQI scores, the subgroup analysis revealed that EA was more effective than Western medication (WMD =-3.598, 95%CI:-4.771 to -2.426), sham acupuncture (SA) (WMD = -3.623, 95% CI: -4.500 to -2.746). For reducing HAMD scores, the subgroup analysis demonstrated that EA had superior efficacy in comparison to Western medication (WMD =-4.903, 95%CI:-6.974 to -2.831), SA (WMD = -4.641, 95% CI: -6.913 to -2.369).

**Conclusion:**

Our findings suggested that EA may be effective in treating depression-related insomnia. However, further large-scale, rigorously designed studies are required to validate its efficacy and safety.

**Systematic review registration:**

https://www.crd.york.ac.uk/PROSPERO/view/CRD42023421281, identifier CRD42023421281.

## 1 Introduction

Depression represents a common psychiatric disorder marked by low mood, loss of interest, and reduced activity due to decreased energy. It stands as one of the most commonly diagnosed mental illnesses. In severe cases, it can lead to suicidal ideation among these patients (1). According to statistics, 13% to 20% of people worldwide have experienced depressive episodes, and the lifetime prevalence of depression varies from 6.1% to 28.2% (2, 3). Global epidemiological surveys indicate that individuals from Southeast Asia and the Western Pacific make up 50% of all depression cases, and Afghanistan is the most severely affected, where over 20% of the population suffers from depression (4). Moreover, the incidence of depression has steadily elevated. From 1990 to 2017, the number of depression cases worldwide rose by 49.86% (5). Depression not only impairs individuals’ daily functioning but also often coexists with other chronic diseases including diabetes and cardiovascular disease. Hence, it can compromise normal bodily functions, reduce quality of life, worsen prognosis, and elevate the death rate in individuals with depression (6).

Insomnia is defined by decreased sleep quality or quantity, frequent nocturnal awakenings, and/or early morning awakening, and difficulty in falling asleep. It may occur as a primary illness or coexist with other mental or physical disorders. About 50% of individuals with depression also suffer from insomnia. Depression and sleep disorders (SD) represent the first and third most common psychological issues encountered in primary healthcare (7). SD often co-occurs with mental illness. As SD are among the most prominent symptoms of depression, they were previously considered a major secondary manifestation of depression. The underlying pathological and physiological mechanisms of their correlation remain inconclusive. Nevertheless, insomnia is considered to be linked to depression, as they frequently co-occur. Recent studies have gradually elucidated the mechanisms linking SD and depression, including the activity of the hypothalamic-pituitary-adrenal axis (8), dysregulation of the 5-hydroxytryptamine (5-HT) system (9), and abnormal expression of inflammatory cytokines (10). These factors contribute to the underlying mechanisms of the comorbidity between depression and insomnia. Therefore, the severity of depression correlates with that of insomnia, with each illness serving as a risk factor for the other (11). In cases where insomnia is present, depression may also be more difficult to treat.

Antidepressants and hypnotics are widely used to treat patients with comorbid depression and SD. Nonetheless, some antidepressants may exacerbate sleep disturbances, and hypnotics may often lead to drug dependence and tolerance. First-line antidepressants include monoamine oxidase inhibitors, serotonin-norepinephrine reuptake inhibitors, selective serotonin reuptake inhibitors, and tricyclic antidepressants (12). However, these medications are linked to adverse effects including tachycardia, dry mouth, blurred vision, constipation, nausea, sedation, and weight gain (12).

Acupuncture (AC) has been widely employed as a complementary therapy in China and other countries (13). Electroacupuncture (EA) integrates traditional AC with modern electrical stimulation. Thanks to its simplicity, safety, and low cost, EA has been extensively used in clinical practice (14). In comparison to other traditional treatments, EA offers advantages such as high reproducibility and standardized frequency, intensity, and duration (15, 16). Moreover, EA is associated with fewer adverse effects compared to pharmacological interventions. When used in combination with medications for managing depression, EA may offer complementary benefits by reducing drug-related side effects and enhancing therapeutic efficacy. In this context, substantial clinical experience has been accumulated in treating SD and depression with EA (17, 18). During EA procedures, fine needles are first inserted into predetermined AC points, after which the output wires of the EA device are connected to the needle handles. The waveform and frequency are then selected according to treatment needs, and the output intensity is gradually increased to a level tolerable to the patient. The primary goal of EA is to enhance the therapeutic effects of manual AC by delivering electrical stimulation through the inserted needles, thereby producing synergistic, excitatory, or inhibitory effects.

Previous studies have indicated that EA is effective in alleviating insomnia related to depression. Studies by Liu C (19) and Yin X (20) unraveled that EA demonstrated more notable efficacy in treating depression-related insomnia than basic treatment and sham acupuncture (SA). However, Yeung WF (21)found no significant difference in efficacy between EA and non-acupoint shallow needling, suggesting that non-specific effects of AC may influence treatment outcomes. Hence, this study seeks to comprehensively assess the clinical efficacy and safety of EA for treating depression-related insomnia.

## 2 Materials and methods

This study was carried out in accordance with the standard methods provided in the Cochrane Handbook. The study protocol was registered in the International Prospective Register of Systematic Reviews (registration number: CRD42023421281).

### 2.1 Literature search

Two reviewers separately searched for studies published up to November 2024 from Embase, China National Knowledge Infrastructure (CNKI), SinoMed, Cochrane Library, Web of Science, PubMed, and Wanfang. The search strategy incorporated both Chinese and English keywords. The Chinese keywords were EA, sleeplessness, insomnia, SD, depression, and depression evidence. The English keywords included EA, depression, sleep initiation, and maintenance disorders. The search strategy is illustrated in **Supplementary Table 1**.

### 2.2 Inclusion and exclusion criteria

The inclusion criteria included: (i) randomized controlled trials (RCTs) assessing the efficacy and safety of EA for treating depression-related insomnia; (ii) participants diagnosed with both insomnia and depressive disorder according to diagnostic criteria including the international classification of diseases [ICD (22)], the Chinese classification of mental disorders [CCMD (23)], the diagnostic and statistical manual of mental disorders [DSM−IV (24) or DSM−V (25)], or the international classification of sleep disorders (ICSD−3) (26), and having a Hamilton depression rating scale (HAMD) score of at least 18 and a Pittsburgh sleep quality index (PSQI) score of at least six, or meeting other equivalent criteria defined by the researchers; (iii) interventions in the experimental group included EA alone or in combination with other treatments (such as conventional pharmacotherapy and auricular plaster therapy). The interventions in the control group included manual AC, SA, or conventional pharmacotherapy. It should be noted that conventional pharmacotherapy encompassed both antidepressants and hypnotics used as control treatments, as well as pre-trial baseline treatments that participants were instructed to continue in some studies, such as oral antidepressants or 23 mg eszopiclone taken as needed for sleep initiation; (iv) studies reported clear outcome measures, such as HAMD score, PSQI score or overall clinical efficacy [the criteria for evaluating clinical efficacy in patients with depression-related insomnia were as follows: clinical recovery was defined as normal sleep duration (>6 hours), deep sleep, refreshed awakening, and ≥75% reduction in HAMD score; effective treatment was defined as symptom relief with sleep duration increased by <3 hours and a reduction in HAMD score of ≥25%; ineffective treatment was defined as no improvement or a reduction in HAMD score of <25% (23)], self−rating depression scale (SDS) score, Hamilton anxiety rating scale (HAMA), insomnia severity index (ISI), one used the self-rating anxiety scale (SAS) and safety outcomes including adverse events and causality assessment, with outcomes evaluated at the end of the intervention phase; (v) no restrictions on case source, disease duration, sex, or age.

The exclusion criteria were: (i) duplicate publications; (ii) studies without a detailed description of the study population, type of study design, and interventions; (iii) letters, reviews, meta−analyses, case reports, expert opinions, editorials, or non−original studies.

### 2.3 Literature screening and data extraction

The literature was separately selected by two authors (GY and LQ) according to the inclusion criteria. Any disagreements were addressed by discussion and, if necessary, uncertainties about study inclusion were resolved by a third reviewer. The extracted data were: (i) basic information: sample size (number of cases in the intervention and control groups), sex, gender, year of publication, and first author’s name; (ii) study characteristics: interventions used in the control and intervention groups, EA frequency, and details of randomization; (iii) outcome measures: evaluation scales, effective rates, and adverse events.

### 2.4 quality assessment

The risk of bias (RoB) tool from the Cochrane Handbook (version 5.0.2) was utilized to evaluate the methodological quality of the included RCTs by two reviewers. Disagreements were addressed by a third reviewer. The assessment criteria included completeness of outcome data, allocation concealment, random sequence generation, selective reporting, blinding of participants and personnel, and other biases. The RoB of each item was classified as low, high, or unclear.

### 2.5 Statistical analysis

Stata SE version 11.0 was employed to carry out statistical analyses. Continuous outcomes were represented by weighted mean differences (WMD) with 95% confidence intervals (CI), while dichotomous outcomes were presented as odds ratio (OR) with 95% CIs. Given the substantial heterogeneity among the controls in the included studies, a random-effects model was applied for the analysis. In this study, considering that the EA intervention group included both EA alone and EA combined with conventional pharmacotherapy, further analyses were conducted to comprehensively assess the treatment effect of EA and ensure the robustness of the findings. Specifically, only the EA groups combined with conventional pharmacotherapy were included in the pooled analysis, in order to assess the consistency and reliability of the findings across different combination therapies. This approach may provide more rigorous evidence supporting the efficacy of EA.

The robustness of our findings was further assessed through a leave-one-out sensitivity analysis. In this approach, each study was sequentially excluded to examine its impact on the pooled estimates. When more than 10 studies were included, Egger’s tests and funnel plots were employed to evaluate publication bias; if fewer than 10 studies were included, only Egger’s test was applied. The trim−and−fill method was employed to adjust for publication bias when Egger’s P < 0.05.

## 3 Results

### 3.1 Study selection

**Figure 1:**
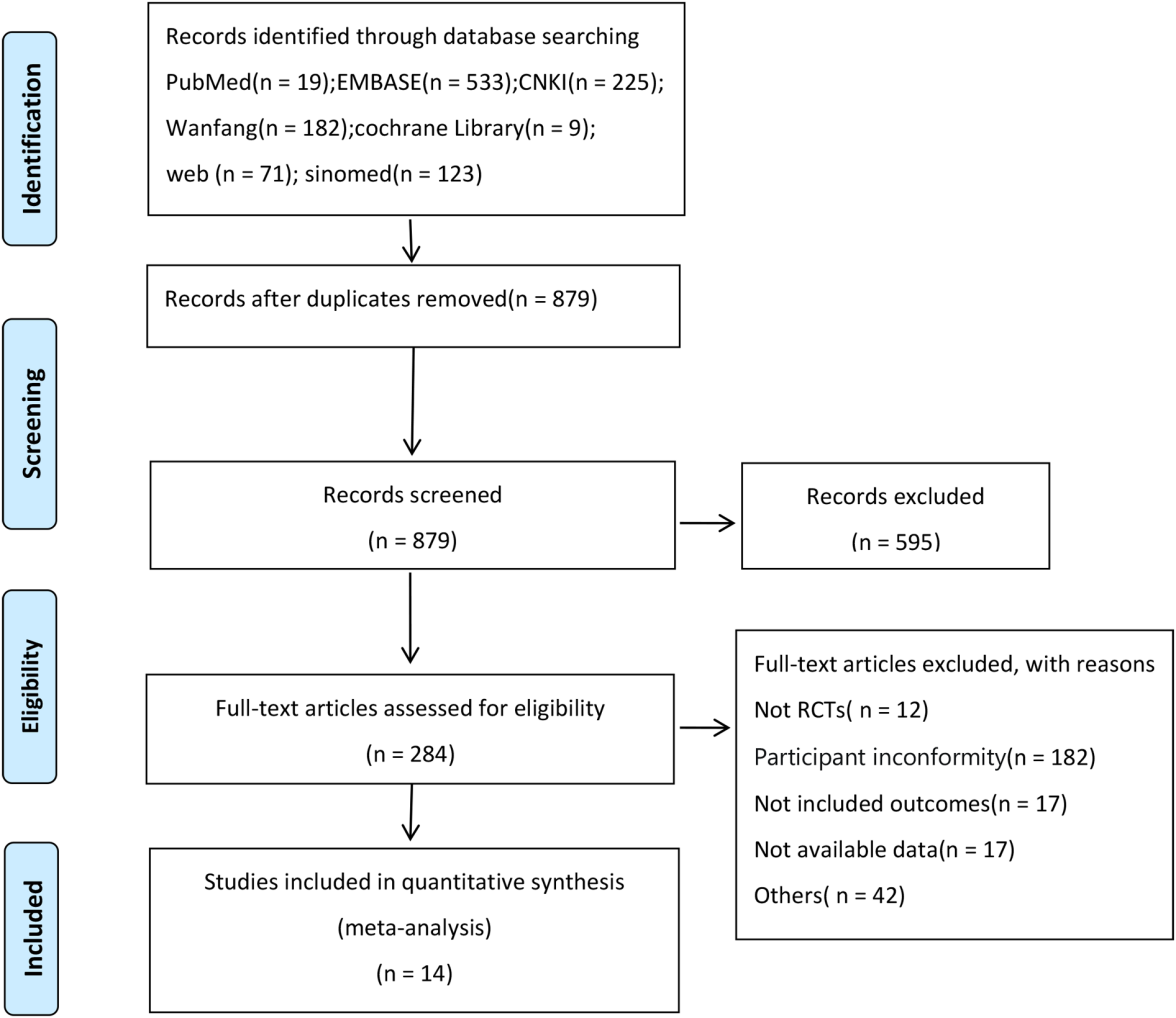
Flow diagram of study screening process.

According to the search strategy, 1162 relevant studies were initially identified. After removing 266 duplicates from different databases, 594 studies were excluded upon reviewing their titles and abstracts. Non-RCTs and RCTs without eligible intervention criteria were further removed after a full-text review. Ultimately, 14 eligible studies (19–21, 27–37)were included. **Figure 1** illustrates the study screening process.

**Table 1** illustrates the characteristics of the eligible RCTs. All included RCTs were published between 2010 and February 2024. 1342 individuals were involved, with 573 in the intervention group and 769 in the control group. 10 RCTs used the PSQI (19–21, 27–33), 14 applied the HAMD (19–21, 27–37), and three used the SDS (27, 29, 34). Additionally, three RCTs employed the HAMA (19, 27, 32), four utilized the insomnia severity index (ISI) (20, 32, 33), one used the self-rating anxiety scale (SAS) (33), six conducted an efficacy analysis (29–31, 34, 36, 37), and five examined adverse reactions after treatment (19–21, 27, 33). Due to the limited number of studies reporting HAMA, ISI, SAS, and SDS outcomes, a meta-analysis of these measures was not feasible. Therefore, this study primarily focused on further analyses based on PSQI, HAMD, treatment efficacy, and adverse event indicators.

**Table 1:** Basic characteristics of the included studies.

Study	Treatment	Sample size	Male/female	Acup	Random method	Acupoints	Electroacupuncture wave forms/frequencies	Duration of electroacupuncture	Outcome
Yin 2022 (20)	EA+CD	90	27/63	50.9 ± 14	Computer software	GV20, GV29	CW	30min	PSQI, ISI, HAMD, AE
	SA+CD	90	30/60	50.5 ± 14					
	CD	90	19/71	49.6 ± 14.9					
Chen 2021 (28)	EA+rTMS+CD	25	16/9	63.44 ± 9.11	NA	GV20, ST36	IW/40Hz	20min	PSQI, HAMD, AE
	rTMS+CD	28	17/11	63.96 ± 7.23					
Han 2019 (34)	EA+CD	25	8/17	37 ± 7.04	Computer software	GV20, EX-HN4, GV29	CW/1Hz	30min	HAMD, SDS
	MA+CD	25	8/17	39 ± 8.52					
Lin 2012 (37)	EA+CD	46	14/32	47 ± 8	The random Number table	GV20, GV29, GB20	DW/2~15 Hz	30min	HAMD
	MA+CD	44	13/31	47 ± 10					
	CD	54	15/39	48 ± 10					
Shi 2013 (30)	EA+CD	38	16/22	57 ± 9	The random Number table	NA	DW	30min	PSQI, HAMD
	CD	38	14/24	55 ± 8					
Su 2013 (29)	MA+APS+CD	32	3/29	43.6 ± 10.97	The random Number table	EX-HN1, EX	CW	30min	PSQI, HAMD, SDS
	EA+APS+CD	28	4/24	48.11 ± 7.3					
	NWM+APS+CD	30	8/22	45.23 ± 10.8					
	MA+CD	27	7/20	46.8 ± 10.46					
	EA+CD	29	10/19	41.97 ± 9.8					
	NWM+CD	31	11/20	44.58 ± 9.3					
Wang 2012 (31)	CD+EA	45	26/19	62.03 ± 4.11	Computer software	GV20, GV29, PC6, HT7, SP6, ST36	CW	30min	PSQI, HAMD
	CD	35	20/15	64.01 ± 4.41					
Xu 2010 (36)	EA+CD	24	10/14	44 ± 12	The random Number table	GV20,GV29	CW/2 Hz	30min	HAMD
	CD	22	10/12	42 ± 15					
	AC+CD	25	11/14	44 ± 13					
Yu 2018 (35)	EA+CD	51	21/20	37.54 ± 9.70	The random Number table	GV20, GV29, EX-HN4, EX	DW/80times/min n~100 times/min	30min	HAMD
	CD	50	23/27	39.4 ± 11.01					
Liu 2021 (19)	EA	29	13/16	47.1 ± 14.08	Computer software	GV20, GV29	CW/2Hz	30min	PSQI, HAMA, HAMD, AE
	SA	27	10/17	45.59 ± 12.6					
Yeung 2011 (21)	EA+CD	26	6/20	47.5 ± 8.5	Computer software	GV29, GV20,SJ21, EX-HN1, EX	NR	30min	PSQI, HAMD, ISI, AE
	SA+CD	26	3/23	50.1 ± 9.1					
Yin 2020 (27)	EA+CD	30	11/19	47.3 ± 14.89	The random Number table	GV20, GV29	CW/30Hz	30min	PSQI. HAMA, HAMD, SDS, AE
	SA+CD	30	10/20	49.8 ± 15.13					
	SA+CD	30	11/19	46.7 ± 15.57					
Yuan 2023 (32)	EA+CD	32	20/12	45.9 ± 14.08	Computer software	GV20, GV29, HT7, SP6	CW/2 Hz	30min	PSQI, SIS, HAMA, HAMD
	SA+CD	30	20/10	45.8 ± 12.67					
Wang 2024 (33)	EA+CD	30	18/12	42 ± 6	Computer software	GV24, GV20, EX-HN1, GV12, ST25, GV6, SP15, GV4, ST36, SP6	CW/30 Hz	30min	PSQI, ISI, HAMD, SAS, AE
	SA+CD	30	17/13	44 ± 6					
	CD	30	16/14	43 ± 6					

EA, electroacupuncture; MA, manual acupuncture; CD, Conventional western medicine; SA, Sham acupuncture; AE, adverse events; NA, Not Applicable; NR, not report; APS, auricular point sticking; NWM, needle-warming moxibustion; CW, continuous wave; IW, intermittent wave; DW, disperse-densewave; GV20, Baihui; GV29, Yintang; ST36, Zusanli; EX-HN4, Taiyang; EX-HN1, Sishencong; EX, Anmian; PC6, Neiguan; HT7, Shenmen; SP6, Sanyinjiao; SJ21, Ermen; GV24, Shenting; GV12, Zhongwan; ST25, Tianshu; GV6, Qihai; SP15, Dahang; GV4, Guanyuan.

Except for one RCT (19) in which the treatment group involved only EA compared with SA, the remaining studies (20, 21, 27–37) combined EA with conventional medication in the treatment groups. Among these, seven RCTs (19–21, 27, 32–34) comparing EA combined with conventional medication with AC, SA, or placebo AC; five studies (30, 31, 35–37) compared EA combined with conventional medication with conventional medication alone; one study (28) compared EA combined with transcranial AC and conventional medication with transcranial AC combined with conventional medication; and one study (29) compared EA and other AC methods, as well as EA combined with auricular therapy versus other AC combined with auricular therapy, all based on conventional medication.

### 3.2 RoB assessment

Among the included studies, five studies were rigorously designed and exhibited low RoB. However, some studies showed a high risk of detection bias due to a lack of blinding and allocation concealment.

**Figure 2:**
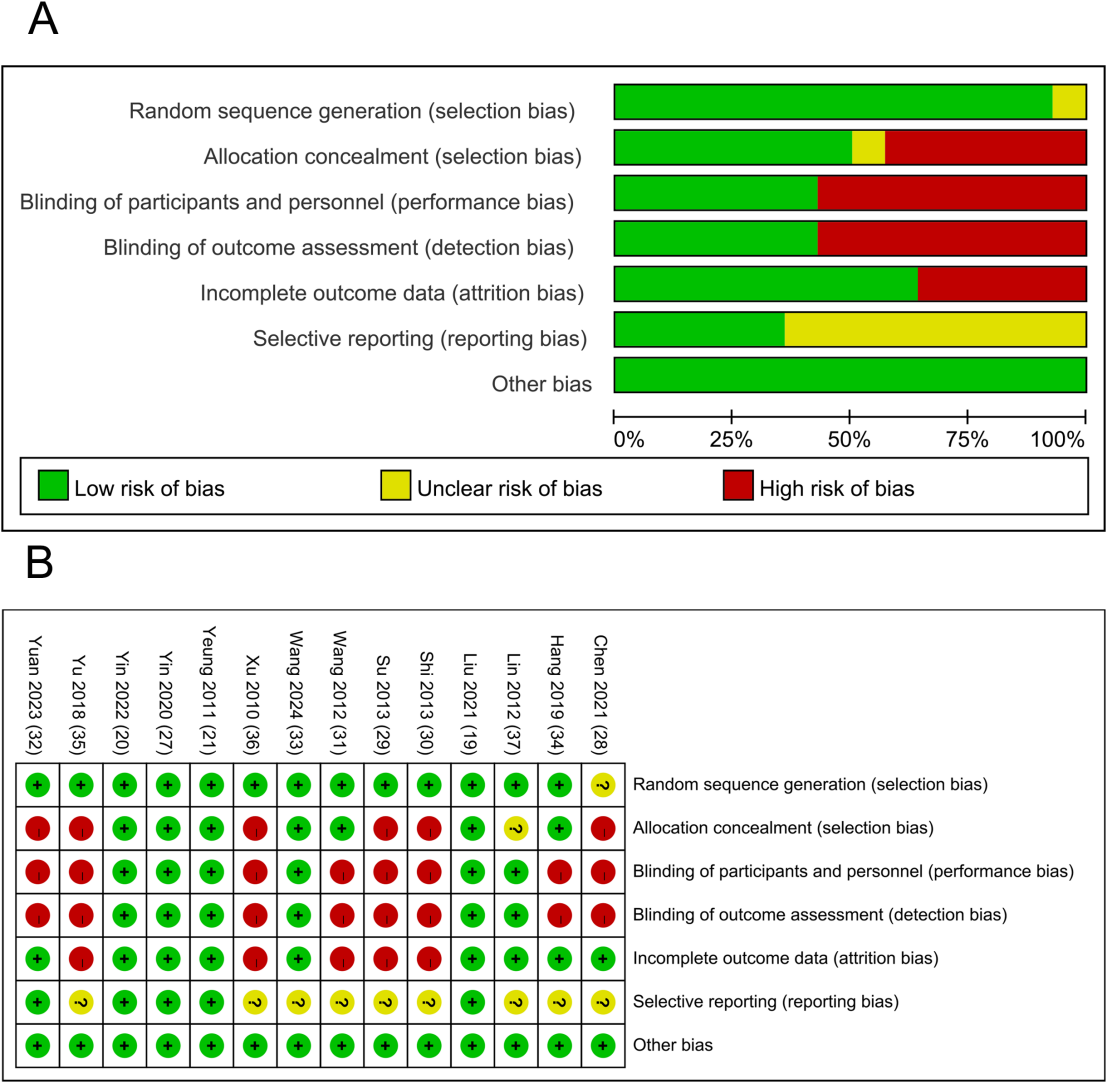
Results of quality assessment of included studies; **(A)** Potential RoB of each included study; **(B)** Summarized RoB of included studies.

Regarding random sequence generation, 13 RCTs (19–21, 27, 29–37) employed appropriate methods, and were thus assessed as having a low RoB. Random sequences were generated using random number tables, computer software, or randomization systems. One RCT (28) did not specify the randomization method. Among them, seven studies (19–21, 27, 31, 33, 34) reported allocation concealment methods, including sealed opaque envelopes (19, 20, 27, 31, 34) and central randomization (21, 33). Six studies (19–21, 27, 33, 37) implemented participant blinding. Six studies (19–21, 27, 33, 37) reported that outcome assessors were blinded to group allocation. Seven studies (19–21, 27, 28, 32, 34) reported dropout cases, with five (19, 21, 27, 34) providing reasons for dropouts. Five studies (19–21, 27, 32) were prospectively registered and had accessible registration records. The assessment results are presented in **Figure 2**.

### 3.3 Meta-analysis results

#### 3.3.1 PSQI score

**Figure 3:**
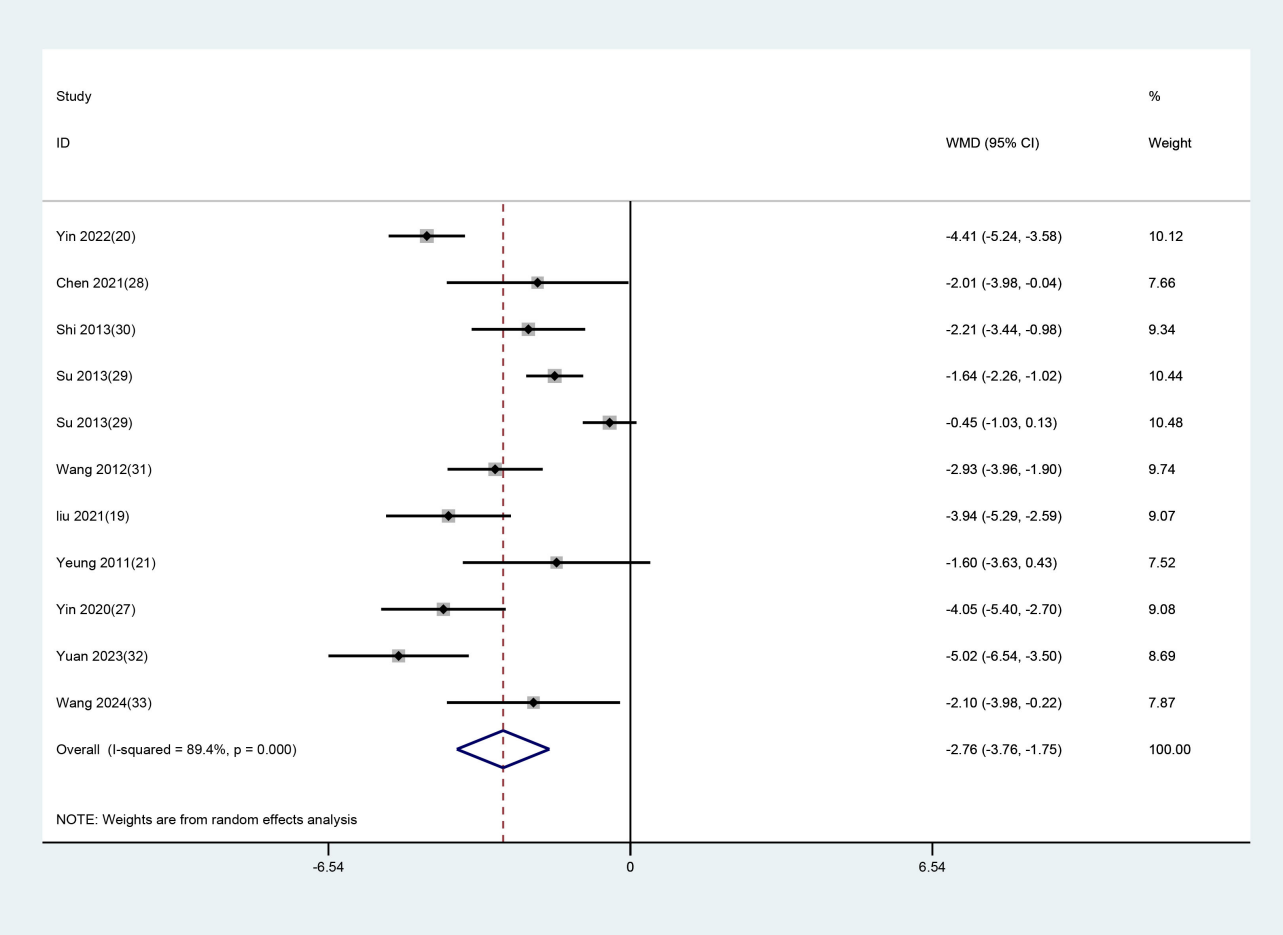
Forest plot and meta-analysis of PSQI assessment scale.

**Figure 4:**
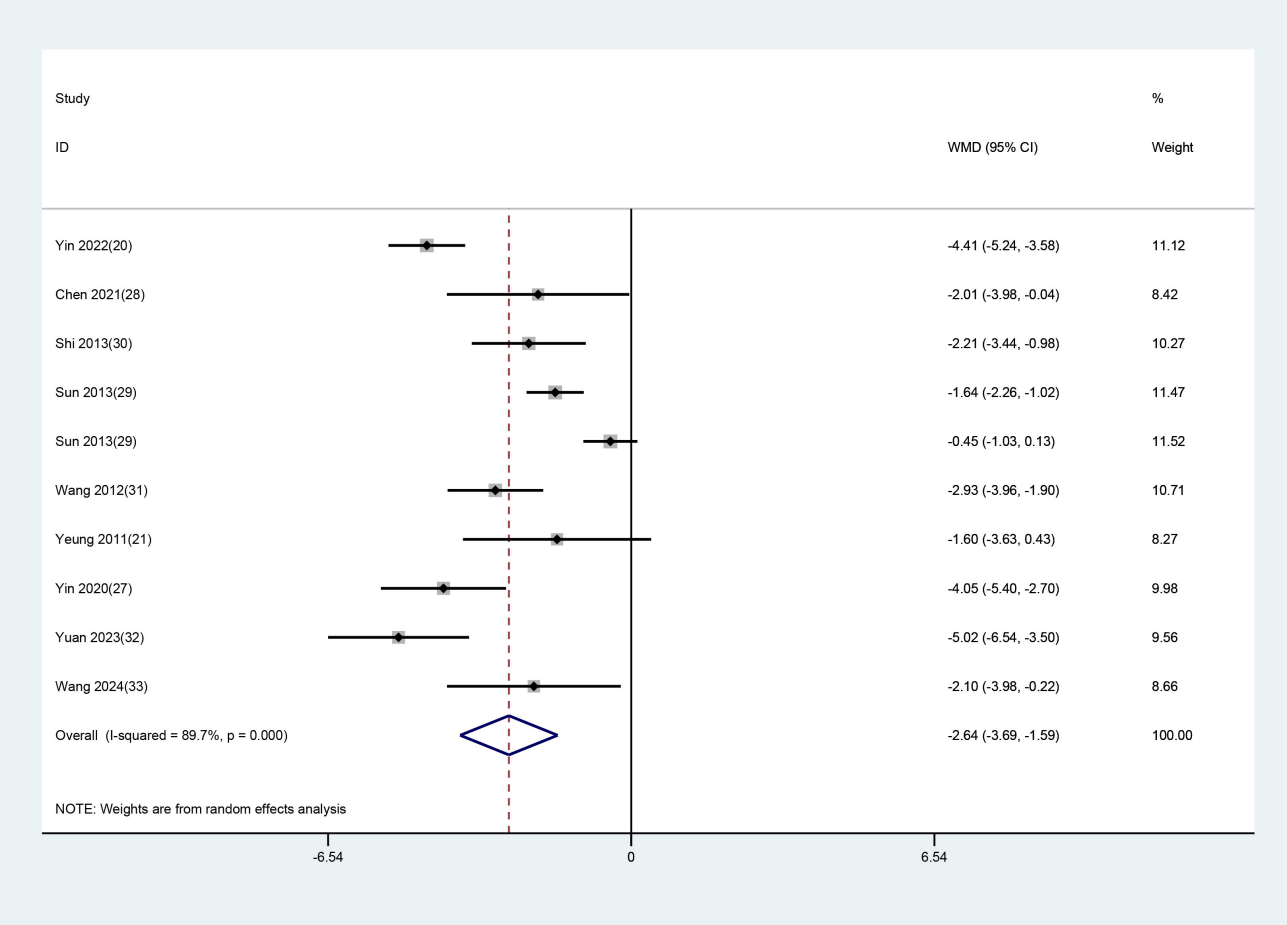
Meta-analysis of PSQI scores for EA combined with conventional medication.

Of the 14 RCTs, 10 studies with 11 datasets used the total PSQI scores after treatment as an indicator of clinical efficacy. As notable heterogeneity (P<0.001, I² = 89.4%) was found, a random-effects model was applied to evaluate the impact of EA (used alone or combined) versus non-EA interventions on PSQI scores. The results unraveled that EA was more effective in lowering PSQI scores in patients with depression-related insomnia than the interventions used in the controls (WMD = -2.757, 95% CI: -3.759 to -1.755). The results are illustrated in **Figure 3**. After excluding one study in which the treatment group received only EA, a further analysis focusing on studies combining EA with conventional medication was performed. The results demonstrated that EA combined with conventional medication was superior to control interventions in reducing PSQI scores in patients with depression-related insomnia (WMD = –2.639, 95% CI: –3.691 to –1.587). These findings are consistent with the previous analyses comparing EA alone or combined with other interventions against non-EA controls. The detailed results are shown in **Figure 4**.

#### 3.3.2 HAMD score

**Figure 5:**
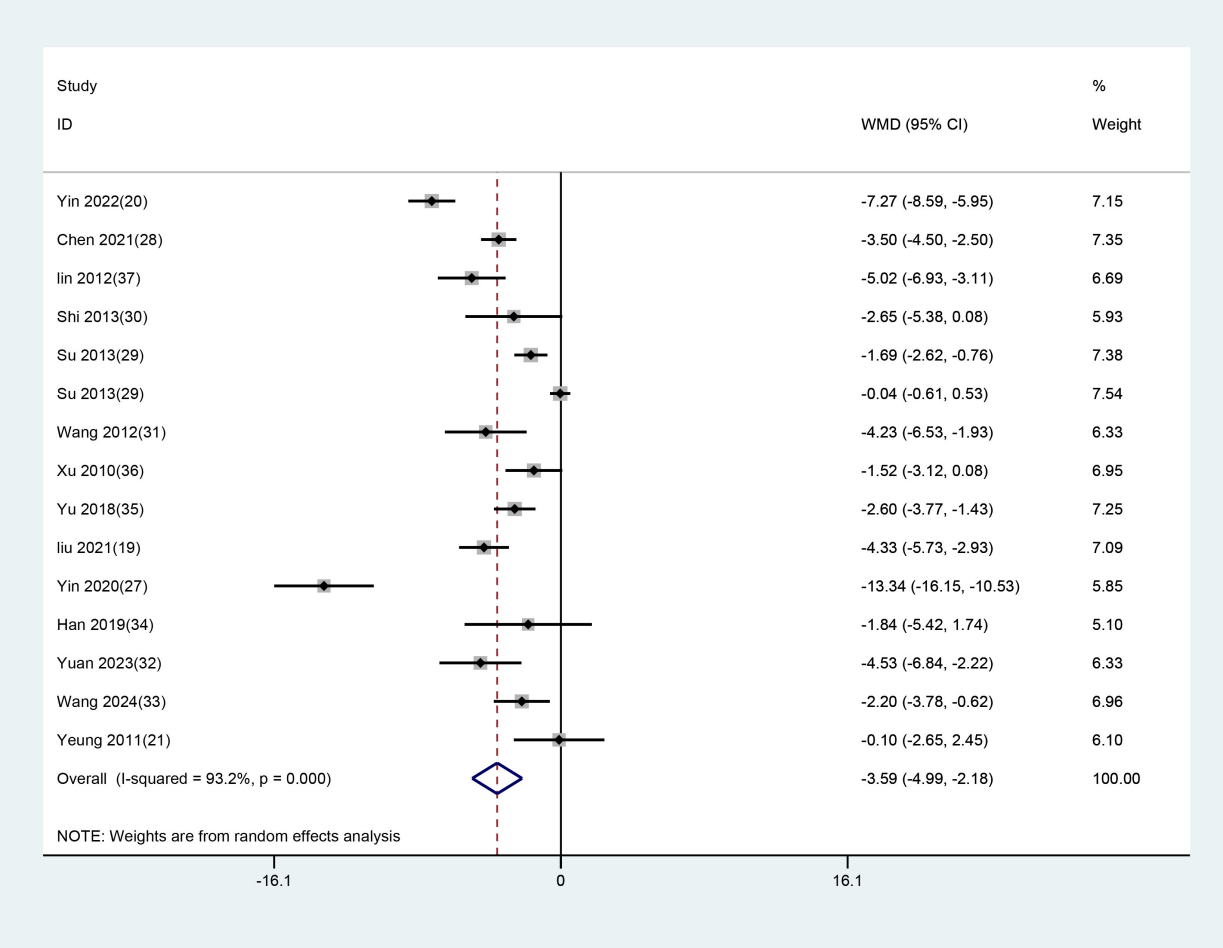
Forest plot and meta-analysis of HAMD assessment scale.

**Figure 6:**
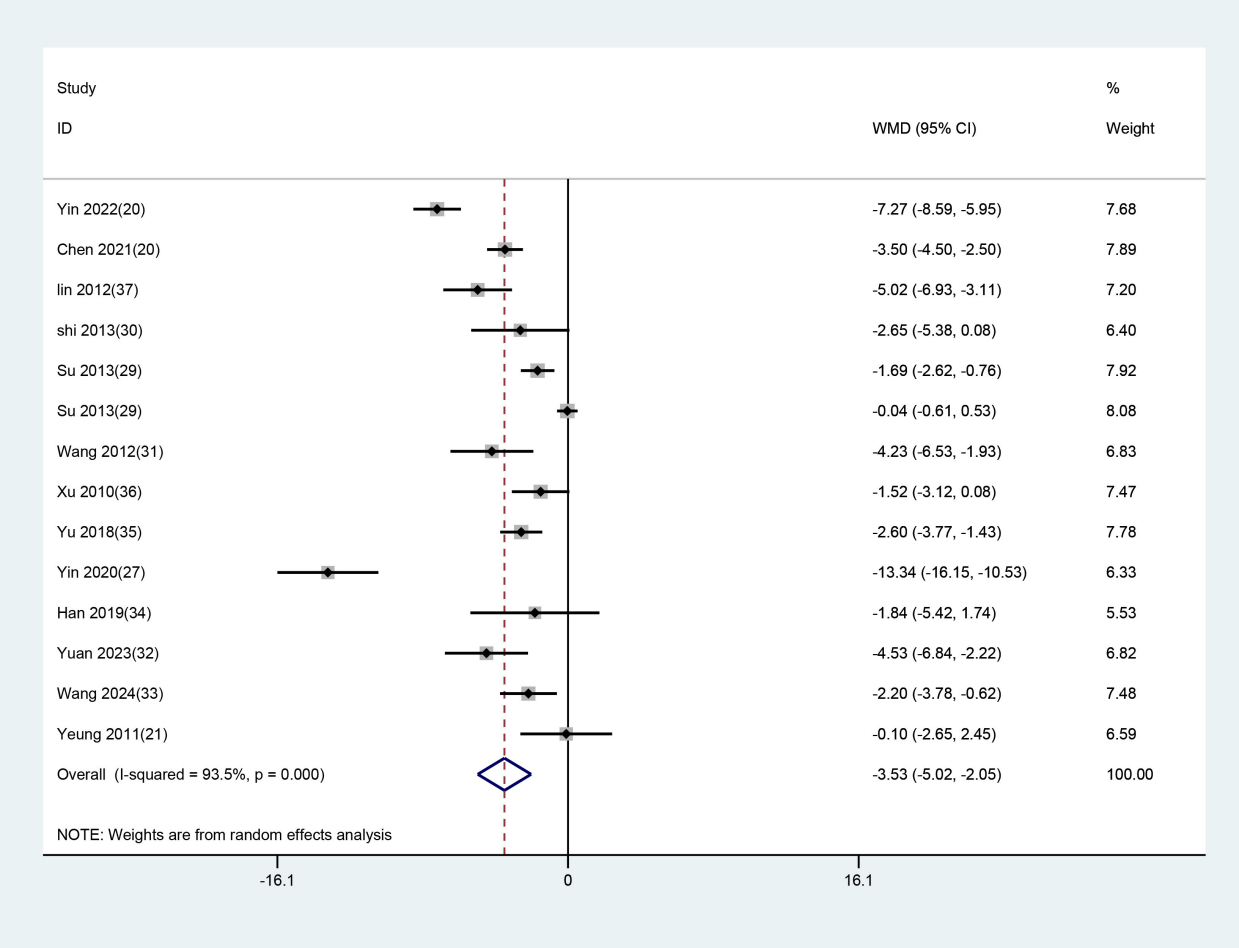
Meta-analysis of HAMD scores for EA combined with conventional medication.

All 14 studies with 15 datasets employed the total HAMD scores after treatment as an indicator of clinical efficacy. As notable heterogeneity (P<0.001, I² = 93.2%) was found, a random-effects model was used to evaluate the impact of EA (used alone or combined) versus non-EA interventions on HAMD scores. The results demonstrated that EA was superior in lowering HAMD scores in patients with depression-related insomnia compared to the interventions used in the control group (WMD = -3.587, 95% CI: -4.992 to -2.182). The results are illustrated in **Figure 5**. After excluding one study in which the treatment group received only EA, a further analysis focusing on studies combining EA with conventional medication was conducted. The results showed that EA combined with conventional medication effectively reduced HAMD scores compared to the controls (WMD = –3.533, 95% CI: –5.016 to –2.050). These findings align with the previous analyses comparing EA alone or combined with other interventions against non-EA controls. The detailed results are presented in **Figure 6**.

#### 3.3.3 Efficacy evaluation

**Figure 7:**
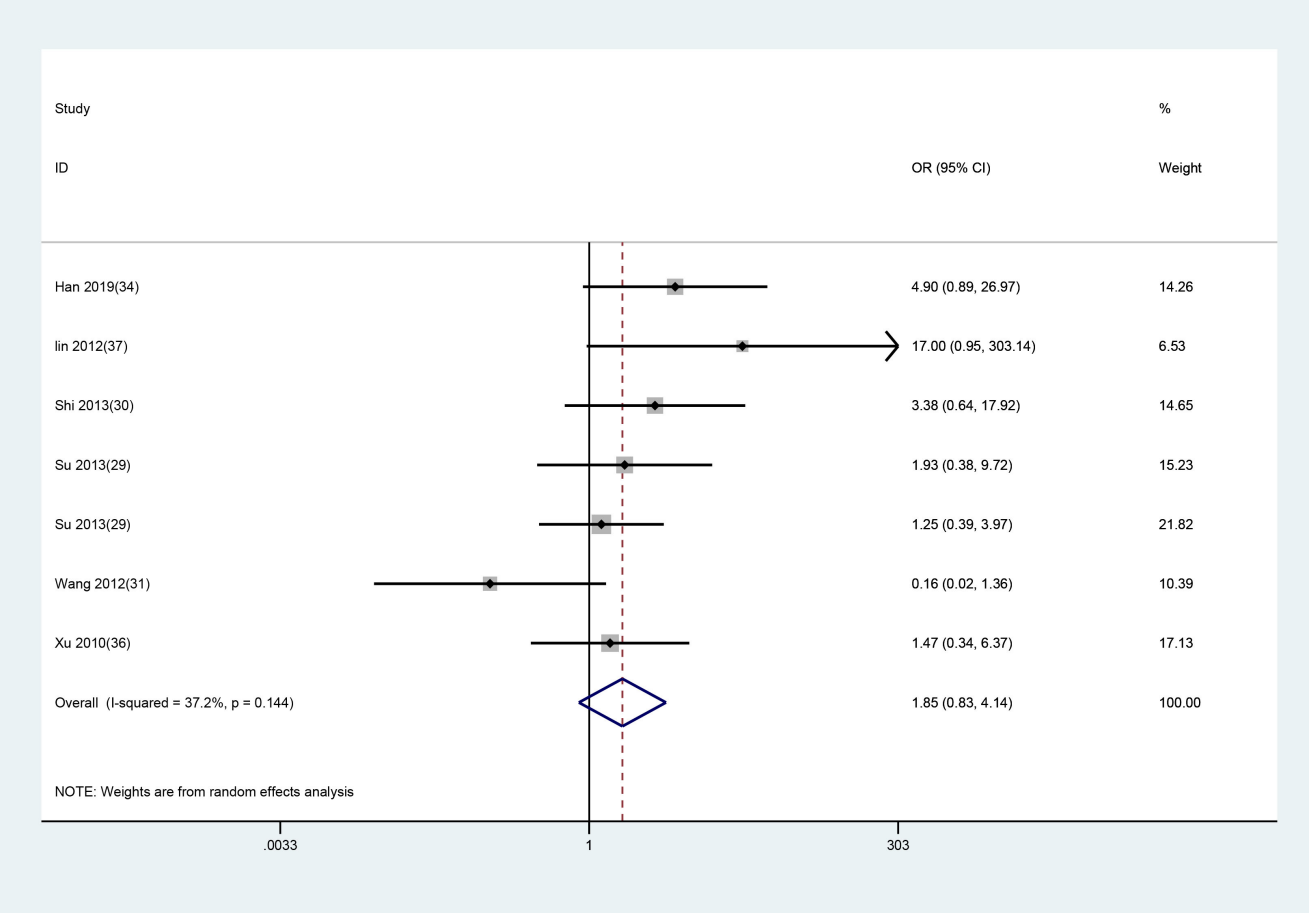
Meta-analysis of the efficacy of EA in treating depression-related insomnia.

Among the 14 included studies, six reported overall treatment efficacy, while the others did not. A random-effects model was applied for analysis, and the results revealed no significant difference in overall efficacy between the EA and control groups (WMD = 1.848, 95% CI: 0.826 to 4.135). The assessment results are presented in **Figure 7**.

#### 3.3.4 Safety evaluation

**Figure 8:**
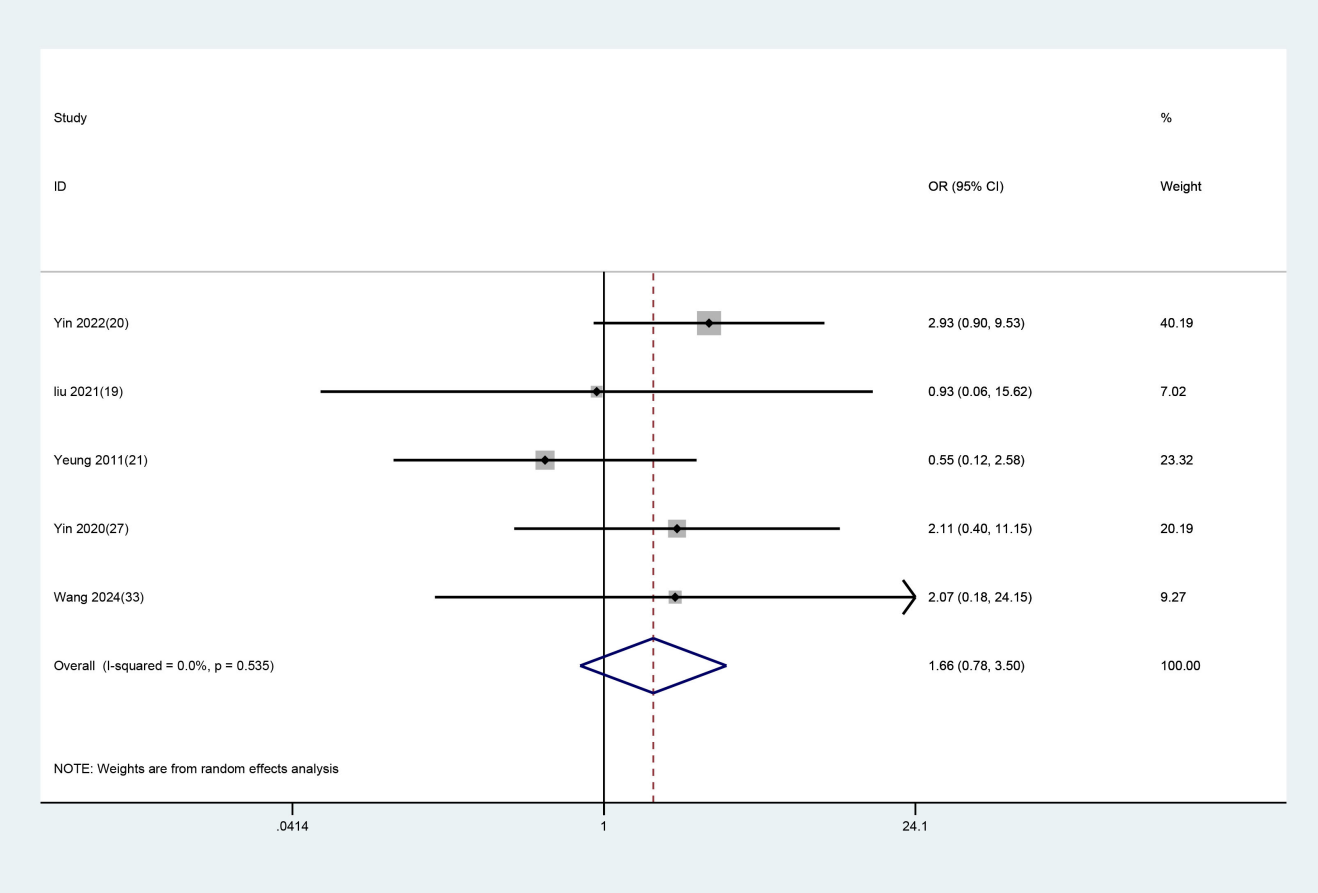
Meta-analysis of the adverse effects of EA in treating depression-related insomnia.

Of the 14 included studies, five reported adverse events, while the others did not. The study by Yin X (20) reported seven cases of subcutaneous hematoma and local pain in the experimental group and four cases of headache in the controls. Liu C (19) reported one case of subcutaneous hematoma in the experimental group and one case of mild fatigue in the control group. Yeung WF (21) reported three cases of headache and dizziness in the experimental group and four cases of dizziness and headache, as well as one case of hand numbness in the control group. Yin X (27) reported three cases of hand numbness and acupoint pain in the experimental group and two cases of dizziness and one case of local hematoma in the control group. Shian Wang XH (33) reported two cases of dizziness and nausea in the experimental group and two cases of fatigue, one case of dizziness, and one case of somnolence in the control group. All adverse events were mild, with no serious adverse reactions reported. A random-effects model was employed for analysis, and the results showed no significant difference in adverse events between the EA and control groups (WMD = 1.657, 95% CI: 0.784 to 3.500). The assessment results are presented in **Figure 8**.

#### 3.3.5 Subgroup analysis

**Figure 9:**
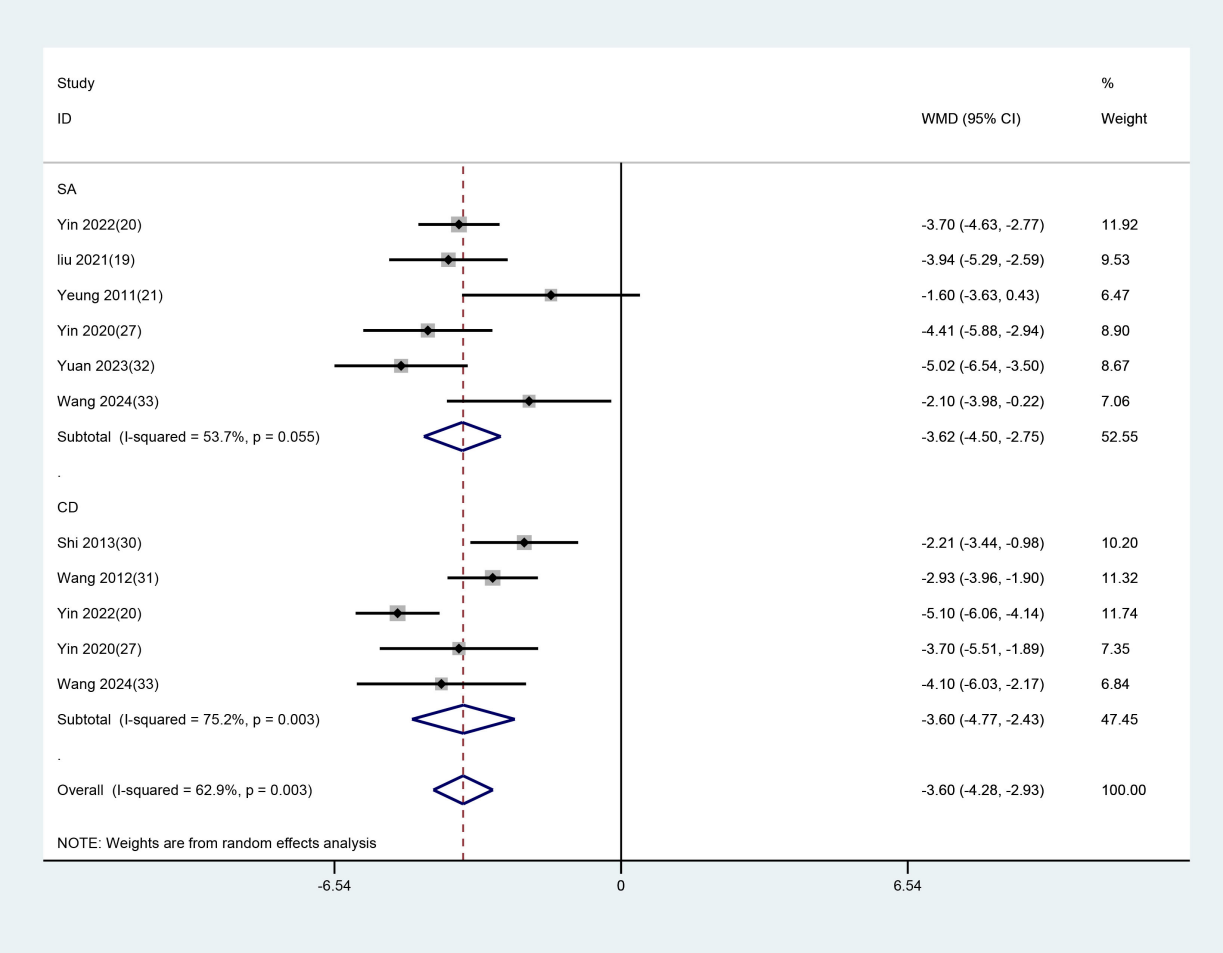
Subgroup analysis of PSQI scores comparing EA with different control interventions. EA, electroacupuncture; SA, sham acupuncture; CD, conventional drugs.

10 studies with 11 datasets used PSQI scores as the outcome indicator. Subgroup analyses were carried out based on different control groups from these studies. Among them, five studies used conventional medication as the control, and the results demonstrated that EA had superior efficacy in contrast to conventional medication (WMD =-3.598, 95%CI: -4.771 to -2.426). Six studies used SA as the control, and the results revealed that EA was more effective than SA (WMD = -3.623, 95% CI: -4.500 to -2.746). (**Figure 9**).

**Figure 10:**
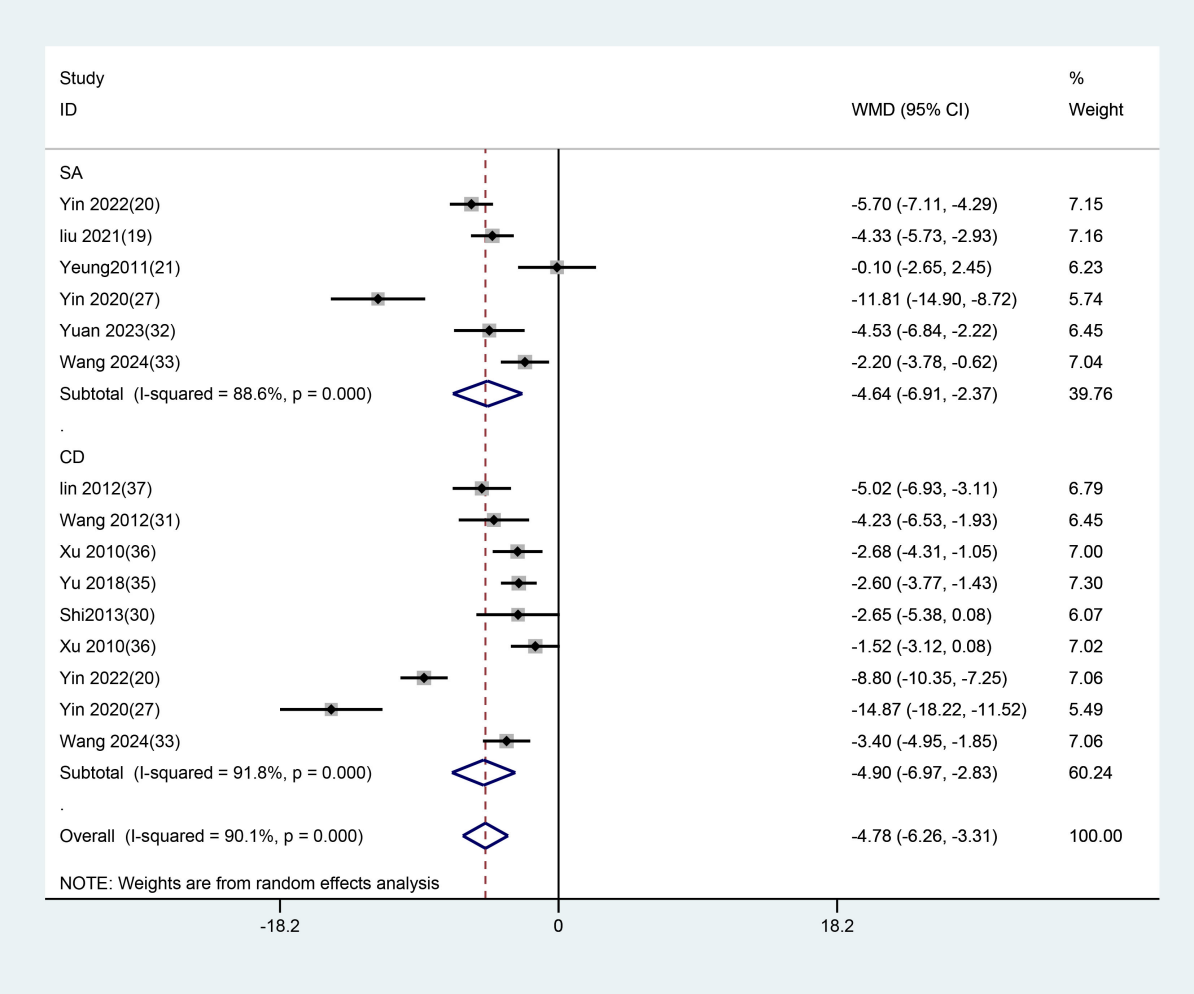
Subgroup analysis of HAMD scores comparing EA with different control interventions. EA, electroacupuncture; SA, sham acupuncture; CD, conventional drugs.

14 studies with datasets applied HAMD scores as the outcome indicator. Subgroup analyses were performed based on different control groups from these studies. Among them, nine studies used conventional medication as the control, and the results demonstrated that EA had superior efficacy in contrast to conventional medication (WMD =-4.903, 95%CI: -6.974 to -2.831). Six studies used SA as the control, and the results demonstrated that EA was more effective than SA (WMD = -4.641, 95% CI: -6.913 to -2.369). (**Figure 10**).

### 3.4 Sensitivity analysis

**Figure 11:**
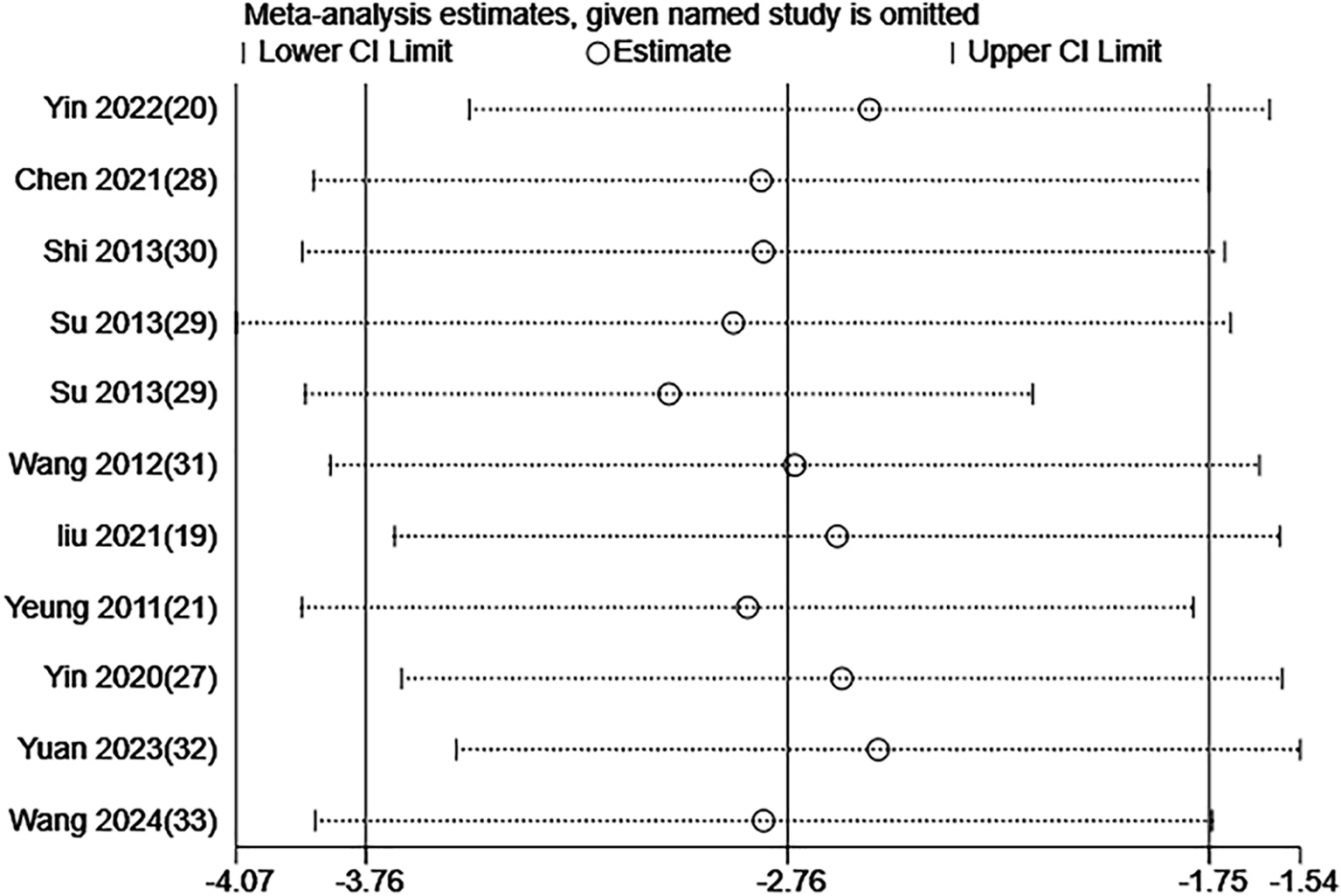
Sensitivity analysis of PSQI score for EA treatment of depression-related insomnia.

**Figure 12:**
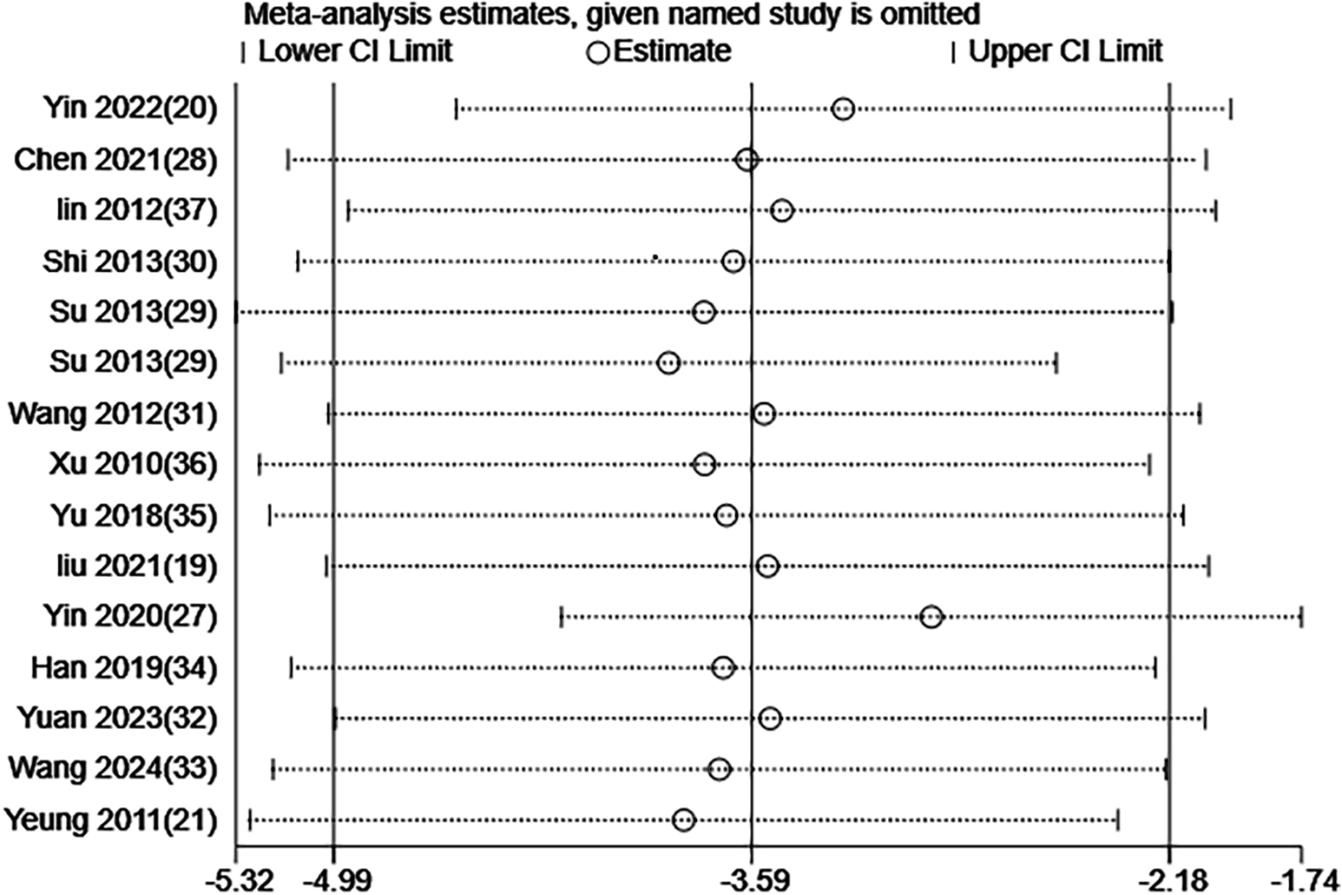
Sensitivity analysis of HAMD score for EA treatment of depression-related insomnia.

The meta-analysis of the scale scores demonstrated high heterogeneity across the included RCTs. Thus, the stability of our findings was examined through a sensitivity analysis via the leave-one-out method. The results demonstrated that removing any single study did not substantially change the overall findings. **Figures 11** and **12** illustrate the results of the sensitivity analysis.

### 3.5 Publication bias

**Figure 13:**
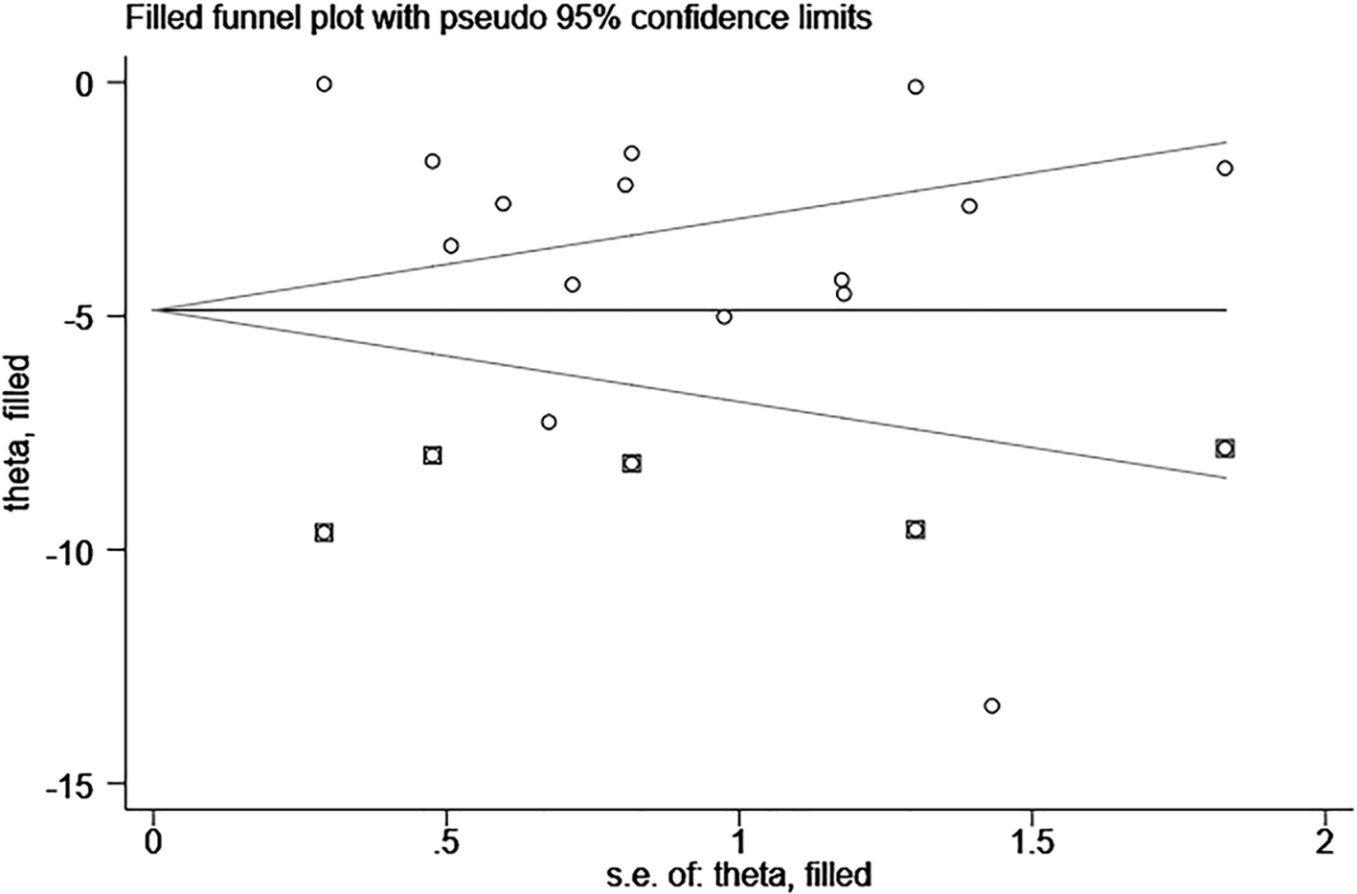
Funnel plot (publication bias) of HAMD score for EA treatment of depression-related insomnia.

Publication bias was assessed using Egger’s test for 11 datasets from 10 studies based on the PSQI and 15 datasets from 14 studies based on the HAMD. No publication bias was detected for PSQI outcomes (P = 0.201). Among the 14 studies reporting HAMD outcomes, a significant difference was observed between the EA and non-EA groups, and Egger’s test indicated potential publication bias (P = 0.023). To further examine and adjust for this bias, the trim-and-fill method was applied. After imputing four hypothetical studies, the adjusted pooled effect size remained significant with a WMD of –4.877 (95% CI: –6.706 to –3.049). These results demonstrated that EA still significantly reduced HAMD scores (**Figure 13**).

## 4 Discussion

This study included 14 RCTs with 1342 individuals to compare the efficacy and safety of EA or combined EA versus non-EA interventions for treating depression-related insomnia. The results unraveled that EA or combined EA substantially decreased PSQI and HAMD scores in contrast to the interventions used in the control group. Subgroup analyses further demonstrated that EA was more effective in mitigating both insomnia and depressive symptoms, regardless of whether it was compared to SA or conventional medication. Nevertheless, since studies reporting adverse events are scarce, there is currently insufficient data to comprehensively examine the safety of EA or combined EA for treating depression-related insomnia.

Three previous meta-analyses investigated AC for treating depression-related insomnia (38–40), including one conducted five years ago (39) and two in recent years (38, 40). Among them, two studies (39, 40) included filiform needle acupuncture (FNA) (or combined AC) and EA (or combined EA) as intervention groups. One study (38) focused solely on FNA and did not analyze the efficacy of EA or combined EA for treating depression-related insomnia. Previous studies have found that AC notably reduces scores on sleep- and depression-related scales. A subgroup analysis in Fei-Yi Zhao’s study (40) reported that EA substantially lowered PSQI and HAMD scores, with effects comparable to conventional medication. Their result is consistent with ours. Our findings indicate that EA significantly improves sleep quality (PSQI: WMD -2.757, 95% CI: -3.759 to -1.755). This PSQI reduction corresponds to approximately 2.75 points, which equates to a decrease in sleep onset time of about 35 minutes (based on PSQI scoring: 1 point ≈ 15-minute decrease in time to fall asleep). In the study by Dong B (39) published in 2017, the effects of EA and SA on reducing PSQI and HAMD scores were compared based on two RCTs published in 2011 and 2015. Their results showed no significant differences between the two groups in either PSQI or HAMD scores. In contrast, our subgroup analysis included six studies (five of which were published within the last five years) and demonstrated that EA, either alone or combined with other treatments, was superior to SA in alleviating depressive symptoms and improving sleep quality. The discrepancy between our findings and those of the previous study may be attributed to the limited number and lower quality of studies included in the earlier analysis, whereas our analysis incorporated more recent and higher-quality evidence.

EA represents an integration of electronic technology with traditional manual AC. It serves as a novel AC-based therapy that retains the therapeutic benefits of traditional AC while incorporating the physiological effects of electrical stimulation. Since EA offers reliable efficacy and provides stable, quantifiable parameters, it is more accessible to patients and is widely used in clinical practice. Similarly, acupoint selection is a key factor influencing the clinical efficacy of AC. Among the studies included in our analysis, the most frequently used acupoints were GV20 and GV29. These acupoints are classic selections for treating mental and psychological disorders. Their hypnotic and antidepressant effects can be explained by traditional Chinese meridian theory and have been validated in both clinical and animal studies. For example, in a sleep-deprived rat model, AC at GV20 regulated cortical excitability, thereby producing a sedative effect (41). A clinical study based on magnetic resonance imaging demonstrated that EA at GV20 and GV29 can exert antidepressant effects by modulating the prefrontal-thalamic circuit (42). In recent years, numerous studies have investigated the efficacy of EA for treating insomnia related to depression. In animal models, EA can regulate excitatory and inhibitory activities in the brain by modulating monoamine neurotransmitters (43), inflammatory cytokines (44), 5-HT, norepinephrine, and hippocampal neurotransmitters and neurons (45), thereby alleviating depressive and sleep-related symptoms. One study included in this meta-analysis (32) reported that EA treatment can notably mitigate depressive symptoms and sleep disturbances, and elevate the levels of serum gamma-aminobutyric acid and serum dopamine. These findings suggest that EA can effectively alleviate depression-related insomnia and its underlying mechanisms involve multi-system, multi-pathway, and multi-target regulation.

In our study, the controls included all non-EA interventions, such as AC, pharmacotherapy, and SA. In addition to an overall evaluation of EA versus non-EA treatments, subgroup analyses based on different control interventions were also carried out. Hence, this study may provide stronger evidence and a more precise assessment of the therapeutic effects of EA. Our study unraveled that EA can be effective in mitigating depression-related insomnia. However, several limitations still exist in this study. First, the methodological quality of the selected RCTs varied. Since most RCTs had small sample sizes and many lacked adequate blinding for outcome assessment, the overall quality of the selected RCTs is low. This may affect the reliability and validity of our findings. Second, the findings may be influenced by the notable heterogeneity in the AC points across the included RCTs. Lastly, the varied duration of treatment across the included studies, ranging from 2 to 8 weeks, may impact the results of our study.

## 5 Conclusion

As a complementary therapy, EA can substantially alleviate depression-related insomnia symptoms. Notably, future studies that investigate the efficacy of EA treatment should improve methodological quality, standardize treatment protocols, and conduct large-scale, multicenter RCTs. This study may lay the foundation for more in-depth research and provide new evidence-based insights into the clinical application of EA.

## Data Availability

The original contributions presented in the study are included in the article/**Supplementary Material**. Further inquiries can be directed to the corresponding author.
