# Supplementary material for: Electroacupuncture for treating depression-related insomnia: a systematic review and meta-analysis
Source: Front Psychiatry. 2025 Jul 8;16:1610107. doi: 10.3389/fpsyt.2025.1610107 (PMC12279889; doi:10.3389/fpsyt.2025.1610107)
Supplement: Supplementary file 1 [file Table1.docx]

**Supplementary Table 1 Search strategy**

**Pub**

| #9 | #8 and #1 | 19 |
| --- | --- | --- |
| #8 | #4 and #7 | 12,187 |
| #7 | #5 or #6 | 561,837 |
| #6 | (((Depressive Symptoms) OR (Depressive Symptom)) OR (Emotional Depression)) | 561,837 |
| #5 | "Depression"[Mesh] | 163,728 |
| #4 | #2 or #3 | 41,666 |
| #3 | ((((((((((((((((((Disorders of Initiating and Maintaining Sleep) OR (DIMS (Disorders of Initiating and Maintaining Sleep))) OR (Early Awakening)) OR (Awakening, Early)) OR (Nonorganic Insomnia)) OR (Primary Insomnia)) OR (Transient Insomnia)) OR (Rebound Insomnia)) OR (Secondary Insomnia)) OR (Sleep Initiation Dysfunction)) OR (Sleep Initiation Dysfunctions)) OR (Sleeplessness)) OR (Insomnia Disorder)) OR (Insomnia Disorders)) OR (Insomnia)) OR (Insomnias)) OR (Chronic Insomnia)) OR (Psychophysiological Insomnia)) | 41,666 |
| #2 | "Sleep Initiation and Maintenance Disorders"[Mesh] | 19,545 |
| #1 | "Electroacupuncture"[Mesh] | 5,407 |

**Embase**

| #5 | #3 AND #4 | 533 |
| --- | --- | --- |
| #4 | #1 AND #2 | 3,882 |
| #3 | 'insomnia'/exp OR insomnia OR agrypnia OR hyposomnia OR (sleep AND initiation AND maintenance AND disorders) OR sleeplessness | 107,157 |
| #2 | 'depression'/exp OR depression OR (central AND depression) OR (clinical AND depression) OR (depressive AND disease) OR (depressive AND disorder) OR (depressive AND episode) OR (depressive AND illness) OR (depressive AND personality AND disorder) OR (depressive AND state) OR (depressive AND symptom) OR (depressive AND syndrome) OR (mental AND depression) OR (parental AND depression) | 1,011,964 |
| #1 | 'electroacupuncture'/exp OR electroacupuncture OR (electrical AND acupoint AND stimulation) OR (acupuncture, AND electric) OR (electrical AND acupuncture) OR 'electro acupuncture' OR (electrode AND acupuncture) OR (electronic AND acupuncture) | 42,039 |

**web**

| #5 | #3 AND #4 | 71 |
| --- | --- | --- |
| #4 | #1 AND #2 | 15,425 |
| #3 | ALL=(Electroacupuncture) | 73,76 |
| #2 | (ALL=(Depression)) OR ALL=((((Depressive Symptoms) OR (Depressive Symptom)) OR (Emotional Depression))) | 764,966 |
| #1 | (ALL=(Sleep Initiation and Maintenance Disorders)) OR ALL=(((((((((((((((((((Disorders of Initiating and Maintaining Sleep) OR (DIMS (Disorders of Initiating and Maintaining Sleep))) OR (Early Awakening)) OR (Awakening, Early)) OR (Nonorganic Insomnia)) OR (Primary Insomnia)) OR (Transient Insomnia)) OR (Rebound Insomnia)) OR (Secondary Insomnia)) OR (Sleep Initiation Dysfunction)) OR (Sleep Initiation Dysfunctions)) OR (Sleeplessness)) OR (Insomnia Disorder)) OR (Insomnia Disorders)) OR (Insomnia)) OR (Insomnias)) OR (Chronic Insomnia)) OR (Psychophysiological Insomnia))) | 50,199 |

**COC**

| #9 | #8 and #1 | 9 |
| --- | --- | --- |
| #8 | #6 and #7 | 2,345 |
| #7 | #4 or #5 | 43,039 |
| #6 | #2 or #3 | 17,676 |
| #5 | (((Depressive Symptoms) OR (Depressive Symptom)) OR (Emotional Depression)) | 31,860 |
| #4 | "Depression"[Mesh] | 18,807 |
| #3 | ((((((((((((((((((Disorders of Initiating and Maintaining Sleep) OR (DIMS (Disorders of Initiating and Maintaining Sleep))) OR (Early Awakening)) OR (Awakening, Early)) OR (Nonorganic Insomnia)) OR (Primary Insomnia)) OR (Transient Insomnia)) OR (Rebound Insomnia)) OR (Secondary Insomnia)) OR (Sleep Initiation Dysfunction)) OR (Sleep Initiation Dysfunctions)) OR (Sleeplessness)) OR (Insomnia Disorder)) OR (Insomnia Disorders)) OR (Insomnia)) OR (Insomnias)) OR (Chronic Insomnia)) OR (Psychophysiological Insomnia)) | 17,082 |
| #2 | "Sleep Initiation and Maintenance Disorders"[Mesh] | 3,875 |
| #1 | "Electroacupuncture"[Mesh] | 1,193 |

**Sinomed**

| #5 | #3 AND #4 | 123 |
| --- | --- | --- |
| #4 | #1 AND #2 | 35,425 |
| #3 | "电针"[常用字段:智能] | 36,894 |
| #2 | "抑郁"[常用字段:智能] OR "郁症"[常用字段:智能] | 715,888 |
| #1 | ("失眠"[常用字段:智能] OR "不寐"[常用字段:智能] OR "睡眠障碍"[常用字段:智能]) | 217,639 |

**知网**

| #5 | #3 AND #4 | 225 |
| --- | --- | --- |
| #4 | #1 AND #2 | 11,300 |
| #3 | （篇关摘：电针(精确)） | 29,200 |
| #2 | （篇关摘：抑郁(精确)）OR（篇关摘：郁症(精确)） | 269,900 |
| #1 | （篇关摘：失眠(精确)）OR（篇关摘：不寐(精确)）OR（篇关摘：睡眠障碍(精确)） | 93,600 |

**万方**

| #5 | #3 AND #4 | 182 |
| --- | --- | --- |
| #4 | #1 AND #2 | 18,599 |
| #3 | （篇关摘：电针(精确)） | 31,835 |
| #2 | （篇关摘：抑郁(精确)）OR（篇关摘：郁症(精确)） | 287,656 |
| #1 | （篇关摘：失眠(精确)）OR（篇关摘：不寐(精确)）OR（篇关摘：睡眠障碍(精确)） | 76,979 |
